# Cytosolic alkalinization in guard cells: an intriguing but interesting event during stomatal closure that merits further validation of its importance

**DOI:** 10.3389/fpls.2024.1491428

**Published:** 2024-11-04

**Authors:** Pulimamidi Bharath, Shashibhushan Gahir, Agepati S. Raghavendra

**Affiliations:** Department of Plant Sciences, School of Life Sciences, University of Hyderabad, Hyderabad, India

**Keywords:** alkalinization, ATPases, ion efflux, secondary messenger, signal transduction, V-ATPase, stomatal closure

## Abstract

Stomatal closure is essential to conserve water and prevent microbial entry into leaves. Alkalinization of guard cells is common during closure by factors such as abscisic acid, methyl jasmonate, and even darkness. Despite reports pointing at the role of cytosolic pH, there have been doubts about whether the guard cell pH change is a cause for stomatal closure or an associated event, as changes in membrane potential or ion flux can modulate the pH. However, the importance of cytosolic alkalinization is strongly supported by the ability of externally added weak acids to restrict stomatal closure. Using genetically encoded pH sensors has confirmed the rise in pH to precede the elevation of Ca^2+^ levels. Yet some reports claim that the rise in pH follows the increase in ROS or Ca^2+^. We propose a feedback interaction among the rise in pH or ROS or Ca^2+^ to explain the contrasting opinions on the positioning of pH rise. Stomatal closure and guard cell pH changes are compromised in mutants deficient in vacuolar H^+^-ATPase (V-ATPase), indicating the importance of V-ATPase in promoting stomatal closure. Thus, cytosolic pH change in guard cells can be related to the rise in ROS and Ca^2+^, leading to stomatal closure. We emphasize that cytosolic pH in stomatal guard cells deserves further attention and evaluation.

## Introduction

The cytosolic pH in plant cells is believed to be relatively stable. However, the available evidences suggest that transient changes in intracellular pH can exert short- and long-term effects. Alkalinization or acidification is often a pre-requisite for plant processes like root hair growth (Monshausen et al., 2007), gravitropism (Felle, 2001), defense responses (Mathieu et al., 1996), phytohormone signaling (Hager, 2003; Li et al., 2022a), and pollen tube elongation (Behera et al., 2018) and stomatal movement (Li et al., 2021; Raghavendra et al., 2023). Changes in intracellular pH are crucial for regulating plant metabolism (Felle, 2001; Zhou et al., 2021; Trinh and Masuda, 2022). As a result, it is debated if cellular pH could be considered a secondary messenger or signaling component, either by itself or along with ROS and Ca^2+^ (Gilroy and Trewavas, 1994; Felle, 2001; Roos et al., 2006).

Stomata regulate the transpirational water loss and restrict the entry of microbial pathogens into leaves. Stomatal opening is induced when guard cells swell due to turgor. Flaccid guard cells shrink and causes stomatal closure. Changes in guard cell turgidity are due to either the loss or accumulation of K^+^, anions (chloride/malate), and organic solutes such as sucrose (Agurla et al., 2018; Yang et al., 2020; Zhang et al., 2024). Whether for opening or closure, guard cell signal transduction ensures ion channels and ion flux modulation, leading to turgor changes. A typical stress hormone, such as abscisic acid (ABA), is sensed and transduced through several signaling components, including receptors, reactive oxygen species (ROS), and cytosolic Ca^2+^. Modulation of these signaling components: ROS, Ca^2+^, and Ca^2+^-dependent protein kinases (CDPK), converge to modulate ion channels and promote ion efflux from guard cells (Kim et al., 2010; Murata et al., 2015; Cotelle and Leonhardt, 2019; Chen et al., 2020; Bharath et al., 2021; Hsu et al., 2021; Liu et al., 2022).

Stomatal movements are associated with pH changes in guard cells (Gonugunta et al., 2009; Islam et al., 2010). However, there has been a debate over the primary importance of cytosolic pH change among the intracellular events leading to stomatal closure. The most intriguing aspect is the relative positioning of pH change with ROS or Ca^2+^ production. Several authors have demonstrated that the pH change preceded ROS or Ca^2+^ production (Irving et al., 1992; Suhita et al., 2004; Islam et al., 2010; Li et al., 2021; Pei et al., 2022; Huang et al., 2023). In contrast, a few reports suggest that cytosolic pH changes were due to elevated ROS/Ca^2+^ (Zhang et al., 2001; Rhaman et al., 2020). In other words, the alkalinization may not always be an early event.

We advocate that the cytosolic pH change can be important in guard cells. While agreeing that cytosolic alkalinization may not be the primary event, we argue that the rise in cytosolic pH in guard cells can promote stomatal closure. We propose an interactive mechanism to explain the argument that pH changes occur either downstream or upstream of ROS or Ca^2+^ rise. Changes in guard cell pH occur during stomatal opening, too, but this aspect has not been much considered in the present article. Similarly, the possible interrelationship of guard cell pH and NO is also not discussed due to the ambiguity of the essentiality of NO for stomatal closure (Ribeiro et al., 2009; Lozano-Juste and León, 2010; van Meeteren et al., 2020).

## Elevation of guard cell pH is typical during stomatal closure

Cytosolic alkalinization precedes the increase in ROS or Ca^2+^ of guard cells during stomatal closure induced by several factors, including hormones, elicitors, and others. Examples are ABA, methyl jasmonate (MeJA), pyrabactin (an analog of ABA), ethylene, sphingosine-1-phosphate (S1P), chitosan, H_2_O_2_, UV-B, and even external Ca^2+^ (**Table 1**). However, the mechanism of how alkalinization could raise ROS or Ca^2+^ levels is not entirely understood. Also, the origin of such pH changes in guard cells too is under debate.

**Table 1:** Elevation of cytosolic pH in guard cells and its consequences on the ROS and Ca^2+^ levels during stomatal closure.

Trigger	Consequence of cytosolic alkalization	Plant	References
Hormones			
Abscisic acid (ABA)	Increase in ROS Increase in ROS followed by Ca^2+^	*Pisum sativum* *Nicotiana tabacum, Arabidopsis* *thaliana*	Gonugunta et al., 2009; Li et al., 2021; Pei et al., 2022
Methyl jasmonate	Elevated ROS	*A. thaliana*	Suhita et al., 2004; Gonugunta et al., 2009
Pyrabactin (ABA analogue)	Increase in ROS	*P. sativum*	Puli and Raghavendra, 2012
Elicitors			
Chitosan	Increased ROS	*P. sativum*	Gonugunta et al., 2009
Yeast Elicitor (YEL)	ROS accumulation	*A. thaliana*	Salam et al., 2013
Others			
Allyl isothiocynate	Elevated ROS, led to rise in cytosolic Ca^2+^	*A. thaliana*	Sobahan et al., 2015; Afrin et al., 2020
Phytosphingosine-1-Phosphate (PhytoS1P)	ROS production and ion channel modualtion	*Vicia faba*	Ma and Niu, 2017
Sphingosine-1-phosphate (S1P)	H_2_O_2_ production	*Vicia faba*	Ma et al., 2012
Darkness	Induced ROS production	*Vicia faba*	Ma et al., 2013
UV-B	Rise in the levels of H_2_O_2_	*A. thaliana*	Zhu et al., 2014
High SO_2_	Increased Ca^2+^ levels	*Tagetes erecta*	Wei et al., 2015
Chloride	Transient alkalinization followed by elevation of cytosolic ABA	*V. faba*	Geilfus et al., 2015
pH modulators			
Methylamine	Induction of H_2_O_2_ production	*A. thaliana*	Zhu et al., 2014
Benzylamine	Mimicked H_2_O_2_ and promoted cytosolic alkalinizations	*V. faba*	Zhang et al., 2001

The changes in cytosolic pH may depend on the vacuolar and other intracellular components. There have been very few reports on the status and pH changes in the vacuole, chloroplast, or other internal membranes of guard cells. The acidic pH of apoplast facilitated stomatal opening, while apoplast alkalinization triggered stomatal closure (Blatt and Armstrong, 1993; Felle et al., 2004; Geilfus, 2017; Inoue and Kinoshita, 2017). The extent of pH change in the cytosol has also been substantial (Ye et al., 2021).

The occurrence of cytosolic pH changes is endorsed by at least three experimental approaches: Modulation of cellular pH by external agents, the use of optimized genetically encoded pH sensors and finally, overexpression/suppression of ATPases. Methylamine and benzylamine (alkalinizing agents) induce stomatal closure in a way similar to ABA or H_2_O_2_, by inducing cytosolic alkalinization followed by H_2_O_2_ production in guard cells (Zhang et al., 2001; Ma et al., 2013; Zhu et al., 2014). In contrast, butyrate or acetate (weak acidifiers), suppress stomatal closure (due to ABA, MeJA, UV-B, H_2_O_2_ or darkness) by reducing cytosolic pH and H_2_O_2_ production in guard cells (Suhita et al., 2004; Islam et al., 2010; Ma et al., 2013; Huang et al., 2014; Zhu et al., 2014).

Most of the pH measurements in plant cells, including guard cells, are made with the pH-sensitive fluorescent dye, 2′,7′-bis-(2-carboxyethyl)-5,(6)-carboxyfluorescein (BCECF) or its membrane-permeant acetoxymethyl ester (BCECF-AM). Recently developed genetically encoded sensors (such as ClopHensor and CapHensor) provide strong evidence that cytosolic pH changes occur along with those of Cl^−^ and Ca^2+^ in guard cells (Arosio et al., 2010; Demes et al., 2020; Li et al., 2021, 2024; Mirasole et al., 2023). Other genetically encoded green fluorescent proteins, including Pt-GFP, pHluorins and At-pHluorins, have demonstrated changes in the cytosolic pH of plant cells (Gao et al., 2004; Schulte et al., 2006; Martinière et al., 2018; Pecherina et al., 2021) but are yet to be tested on guard cells. These recent pH sensors can monitor cytosolic pH and ions such as Ca^2+^ or chloride in real time, thus providing an advantage in measuring pH and ion dynamics.

External agents such as methylamine/benzylamine provide indirect evidence of pH changes. So far most of the pH changes in guard cells are monitored by using fluorescence dye, BCECF-AM. However, doubts are expressed about the preciseness of BCECF-AM. Recent studies with advanced pH sensors indicate that elevation of pH changes can occur as early as 2 mins followed by Ca^2+^/ROS changes (Li et al., 2024). Arabidopsis mutants deficient in H^+^-ATPases (PM-/V) also could be important for asserting their involvement during stomatal closure. Among these, advanced pH sensors and the use of ATPase mutants can provide convincing evidence of cytosolic pH changes during stomatal closure.

Changes in guard cell pH can occur when ATPases are modulated. This aspect is discussed in the next section.

## The origin of pH-rise in guard cells: Involvement of vacuolar-ATPases

Stomatal opening is restricted when plasma membrane-ATPase (PM-ATPase) is inhibited (Takemiya and Shimazaki, 2010). Upregulation of PM H^+^-ATPase activity appears to be necessary for stomatal opening. However, the role of PM-ATPase during stomatal closure is ambiguous. Two dominant mutations in the *open stomata 2* (*OST2*) gene result in constitutive activation of AHA1 (gene encoding PM-ATPase), abolishing ABA-induced closure and keeping stomata open (Merlot et al., 2007). Stomatal closure by ABA is compromised in loss-of-function mutants of *aha2-6* and *aha2-6 bak1-4* double mutants (Pei et al., 2022). Thus, the role of PM-ATPase during stomatal closure is confusing, and a question arises if the two forms of AHA1 and AHA2 act differently. Further work is needed to establish if PM-ATPase has a dual role during stomatal opening or closure.

On the other hand, there is strong evidence for the role of vacuolar H^+^-ATPase (V-ATPase) mediated vacuolar acidification and cytosolic alkalinization during stomatal closure. Alkalinization of guard cells by H_2_O_2_ or phosphatidylinositol 3,5 bisphophate [PI(3,5)P2] is due to H^+^-efflux from the cytosol into the vacuole involving V-ATPase (Zhang et al., 2001; Bak et al., 2013). Suppression of V-ATPase (as in *vha-a* mutant) results in enhanced stomatal aperture (Zhang et al., 2013). Arabidopsis V-ATPase double mutant (*vha-a2 vha-a3*) has no vacuolar H^+^-pumping activity and exhibits delayed vacuolar acidification and annulled stomatal closure in response to ABA (Bak et al., 2013). Down-regulation of phosphatidylinositol3-kinase (*pi3k*), a protein kinase that activates V-ATPase, results in low vacuolar acidification and limited stomatal closure in response to MeJA (Liu et al., 2016). A deficiency of V-ATPase (as in *de-etiolated-3*/*det3* mutant) or RNAi interference, results in enhanced opening (Allen et al., 2000; Zhang et al., 2013; Seidel, 2022). Thus, vacuolar acidification was closely associated with cytosolic alkalinization.

5-aminolevulinic acid, a potential plant growth regulator, promotes stomatal opening and reverses ABA-induced closure by downregulating V-ATPase and restricting guard cell pH and H_2_O_2_ levels in apple leaves (Hu et al., 2019). Cytosolic pH and ROS levels are low in several of these instances. An active V-ATPase can cause cytosolic alkalinization and raise H_2_O_2_ levels in guard cells during stomatal closure. Besides V-ATPase, vacuolar-PPase (V-PPase) can cause rapid acidification of vacuoles during stomatal closure induced by ABA (Eisenach and De Angeli, 2017). But, the specific role of V-PPase needs to be examined in detail.

## Discussion

### Cytosolic alkalinization in relation to the scheme of signaling events during stomatal closure

Stomata close when guard cells lose their K^+^/Cl^-^ triggered by an increase in intracellular Ca^2+^ of guard cells. Whenever the stomata are exposed to biotic/abiotic stress signals, the levels of two major secondary messengers, ROS and Ca^2+^, increase in guard cells. The perception of a signal such as ABA (a plant hormone), or flagellin (microbial elicitor) activates OST1 kinase and NADPH oxidase to promote H_2_O_2_ production. The elevated ROS initiates the efflux Ca^2+^ from endo-cytomembranes, an influx of external Ca^2+^, or both. This scheme of signaling events during stomatal closure is well accepted (Bharath et al., 2021; Hsu et al., 2021; Meddya et al., 2023; Zhang et al., 2024).

The temporal studies indicate that the increase in guard cell pH is the earliest, followed by ROS or Ca^2+^ (Suhita et al., 2004; Ma et al., 2012; 2013; lozanoZhu et al., 2014). Using genetically encoded pH/Ca^2+^ sensor, Li et al. (2021) have observed that ABA elevated cytosolic pH by ~2 min, followed by Ca^2+^ in >5 min. However, the mechanism of pH-induced ROS production in guard cells has yet to be elucidated. One of the possibilities is that alkalinization and subsequent release of Ca^2+^ (from endo-cytomembranes) could facilitate the activation of NADPH oxidase through the Ca^2+^-dependent phosphorylation of SnRK-type OST kinase (Han et al., 2019; Kimura et al., 2022; Liu et al., 2022).

**Figure 1:**
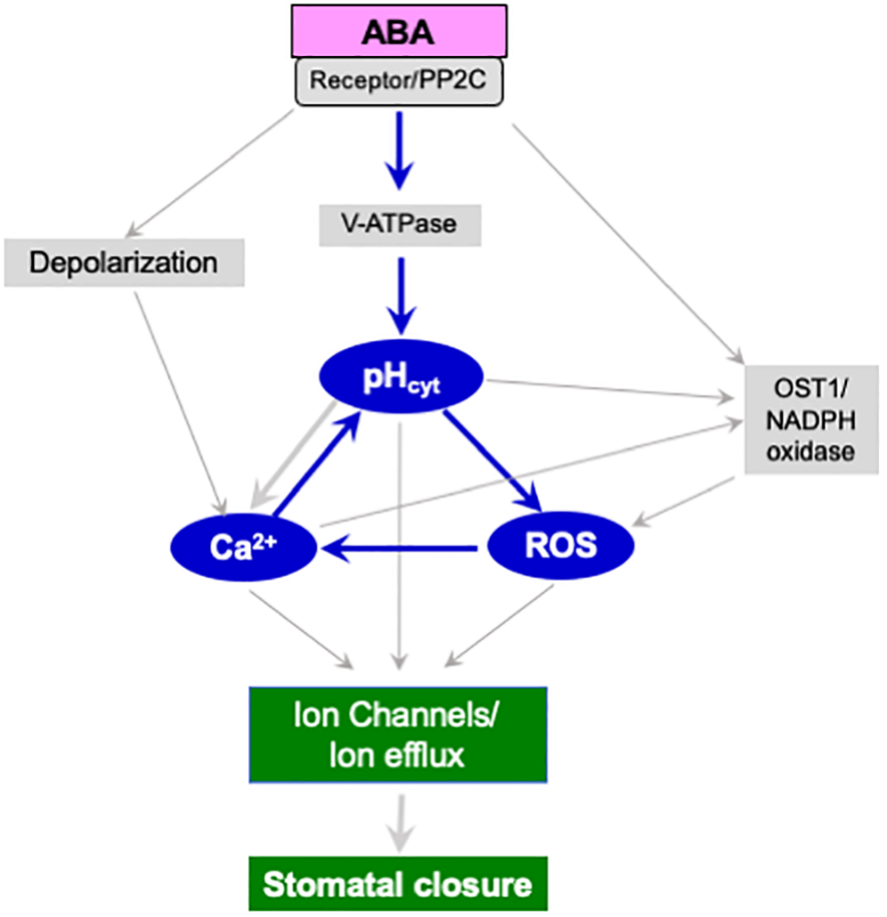
A hypothetical scheme of signaling components participating in stomatal closure induced by ABA. Changes in the cytosolic pH (pH_cyt_) of guard cells can modulate ROS and cytosolic Ca^2+^. The cytosolic alkalinization caused by ABA seems to be due to the activation of vacuolar H^+^-ATPase (V-ATPase). The stomatal closure by ABA involves the binding of ABA to its receptor (PYR/PYL/RCAR), inhibition of protein phosphatase (PP2C), and activation of protein kinase (OST1). In turn, OST1 kinase activates NADPH oxidase to produce ROS. Elevated ROS can increase cytosolic Ca^2+^ levels and directly affect ion channels like SLAC1 (Gilroy et al., 2016; Liu et al., 2022). Parallelly, cytosolic alkalinization promotes membrane depolarization, increased Ca^2+^ and a rise in pH. At the same time, elevated pH, ROS, and Ca^2+^ upregulate outward channels of K^+^, Cl^ˉ^, and NO_3_^ˉ^, causing a net efflux of ions, loss of guard cell turgor, and stomatal closure. These components may all interact. As per our feedback activation model (indicated in blue), adding H_2_O_2_, or Ca^2+^, can promote the rise in pH and *vice-versa*. The published evidence endorses the increase in ROS by guard cell alkalinization (Islam et al., 2010), the elevation of Ca^2+^ by ROS (Liu et al., 2022), the rise in pH by Ca^2+^ (Li et al., 2021; Huang et al., 2023). The upregulation of pH by Ca^2+^ is also known (Islam et al., 2010).

The secondary messengers, ROS and Ca^2+^, may act either upstream or downstream of cytosolic alkalinization in an interactive manner to promote ion efflux and stomatal closure (**Figure 1**). In addition to ROS or Ca^2+^, other signaling molecules that can induce cytosolic pH changes include ethylene, S-1-P/phyto S-1-P (**Table 1**) and PI(3,5)P2 (Bak et al., 2013). However, their action seems to converge at ROS or Ca^2+^ or both. Further studies are needed to identify the exact conditions when alkalinization precedes or co-occurs with ROS generation. Parallelly, the cytosolic pH can directly modulate the outward K^+^-channels and promote K^+^ efflux from guard cells (Blatt, 1990; Blatt and Armstrong, 1993; Grabov and Blatt, 1997). In addition, the modulation of OST1 kinase/NADPH oxidase/ROS/ion channels and increased ion flux leading to stomatal closure can also occur independent of cytosolic pH change.

## Ambiguities to be resolved

Changes in guard cell pH can be mediated by the membrane potential and ion fluxes and *vice versa*. During stomatal closure, the cytosolic pH increases, followed by membrane depolarization and increased K^+^/Clˉ efflux from guard cells (Blatt and Armstrong, 1993; Miedema and Assmann, 1996; Pandey et al., 2007; Chen et al., 2020). Membrane depolarization and Ca^2+^ influx can modulate pH in plant cells, including guard cells (Brault et al., 2004; Meimoun et al., 2009; Pottosin et al., 2014). As per Roelfsema et al. (2004), ABA can cause membrane depolarization and activation of outward ion channels. Such a situation still does not decrease the importance of cytosolic alkalinization during stomatal closure.

ATPases, particularly PM-ATPase and V-ATPase are among the most important proteins that can modulate intracellular pH (Roelfsema and Hedrich, 2005; Kim et al., 2010). Ion-transporters (particularly Ca^2+^, Cl^-^ or NO_3_) and CONSTITUTIVE PHOTOMORPHO-GENIC 1 (COP1, a light-sensitive, negative regulator of stomatal opening) can also drive pH-changes in guard cells to modulate stomatal movement (Eisenach and De Angeli, 2017; Demes et al., 2020; Dreyer et al., 2022; Li et al., 2024; Cha et al., 2024). A few other proteins, such as cation/H^+^ transporters and transcription factors (PacC, a dominant transcription factor), are known to modify the cellular pH, but their role in guard cells is uncertain (Pittman and Hirschi, 2016; Li et al., 2022b). However, ion-transporters’ role in modulating guard cell pH is unclear and needs further study.

Another criticism is that the rise in pH may not all be cytoplasmic. The dye BCECF-AM, with a pKa value of 6.98, is expected to stay within the cytosol (Boyer and Hedley, 1994; Paradiso et al., 1984). Further, BCECF-AM has been widely used to detect the pH changes in root hairs and pollen tissues besides guard cells (Gehring et al., 1990; Kosegarten et al., 1997; Han and Burgess, 2010; Wilkins et al., 2015; Yemelyanov et al., 2020). We, therefore, believe that the elevated fluorescence by BCECF-AM upon ABA or MeJa treatment originates mainly from the cytosol of guard cells. An additional confusion arises when the buffer strength of cellular components is considered. The buffer strength of cytosol is expected to be several times that of apoplast. However, Oja et al. (1999) have suggested that fast cytoplasmic pH changes can occur due to the pumping of protons into the vacuole. Using genetically encoded sensors that are much more robust than the fluorescent dyes also validates the cytosolic alkalinization in guard cells during closure (see Section 2).

## Future perspectives

The importance of guard cell pH in mediating stomatal closure cannot be ignored. We emphasize that the guard cell pH can be an important event that modulates stomatal movements, even if the pH change in guard cells is not necessarily the primary cause. Other aspects that need critical re-evaluation in guard cells are pH changes in different intracellular compartments, the exact values of cytosolic pH and time-dependent dynamics of pH change. The relationship between changes in pH, ROS and Ca^2+^ can vary depending on the trigger, for e.g. ABA or flagellin (Li et al., 2021). The availability of genetically encoded dual pH, Ca^2+^ or K+ sensors would be extremely useful in resolving some of these issues.

It is quite fascinating to consider the possible mechanism of “pH-sensing” in guard cells. The occurrence of pH sensors in plant cells is often discussed, but the mechanism of pH sensing is still unclear (Li and Yang, 2023). Among the possible molecules that could be relevant to guard cells are phosphatidic acid (Li et al., 2019), PP2C D-clade proteins (Wong et al., 2019) and vacuolar transporters (Demes et al., 2020). We expect our article on guard cell pH will trigger further research into this intriguing but fascinating topic of cytosolic pH as a key event. Stomatal guard cells are promising model systems for examining pH’s role in plant tissues.

## References

[B1] Afrin S. Okuma E. Tahjib-Ul-Arif M. Jahan M. S. Nakamura T. Nakamura Y.. (2020). Stomatal response to isothiocyanates in *Arabidopsis thaliana* . J. Exp. Bot. 71, 6921–6931. doi: 10.1093/jxb/eraa420 33252127

[B2] Agurla S. Gahir S. Munemasa S. Murata Y. Raghavendra A. S. (2018). Mechanism of stomatal closure in plants exposed to drought and cold stress. Adv. Exp. Med. Biol. 1081, 215–232. doi: 10.1007/978-981-13-1244-1_12 30288712

[B3] Allen G. J. Chu S. P. Schumacher K. Shimazaki C. T. Vafeados D. Kemper A.. (2000). Alteration of stimulus-specific guard cell calcium oscillations and stomatal closing in *Arabidopsis det3* mutant. Science 289, 2338–2342. doi: 10.1126/science.289.5488.2338 11009417

[B4] Arosio D. Ricci F. Marchetti L. Gualdani R. Albertazzi L. Beltram F. (2010). Simultaneous intracellular chloride and pH measurements using a GFP-based sensor. Nat. Methods 7, 516–518. doi: 10.1038/nmeth.1471 20581829

[B5] Bak G. Lee E. J. Lee Y. Kato M. Segami S. Sze H.. (2013). Rapid structural changes and acidification of guard cell vacuoles during stomatal closure require phosphatidylinositol 3,5-bisphosphate. Plant Cell 25, 2202–2216. doi: 10.1105/tpc.113.110411 23757398 PMC3723621

[B6] Behera S. Zhaolong X. Luoni L. Bonza M. C. Doccula F. G. De Michelis M. I.. (2018). Cellular Ca^2+^ signals generate defined pH signatures in plants. Plant Cell 30, 2704–2719. doi: 10.1105/tpc.18.00655 30377237 PMC6305977

[B7] Bharath P. Gahir S. Raghavendra A. S. (2021). Abscisic acid-induced stomatal closure: An important component of plant defense against abiotic and biotic stress. Front. Plant Sci. 12. doi: 10.3389/fpls.2021.615114 PMC7969522 33746999

[B8] Blatt M. R. (1990). Potassium channel currents in intact stomatal guard cells: rapid enhancement by abscisic acid. Planta 180, 445–455. doi: 10.1007/BF00198799 24202027

[B9] Blatt M. R. Armstrong F. (1993). K^+^ channels of stomatal guard cells: abscisic-acid-evoked control of the outward rectifier mediated by cytoplasmic pH. Planta 191, 330–341. doi: 10.1007/BF00195690

[B10] Boyer M. J. Hedley D. W. (1994). Measurement of intracellular pH. Methods Cell Biol. 41, 135–148. doi: 10.1016/S0091-679X(08)61714-8 7532259

[B11] Brault M. Amiar Z. Pennarun A. M. Monestiez M. Zhang Z. Cornel D.. (2004). Plasma membrane depolarization induced by abscisic acid in Arabidopsis suspension cells involves reduction of proton pumping in addition to anion channel activation, which are both Ca^2+^ dependent. Plant Physiol. 135, 231–243. doi: 10.1104/pp.104.039255 15141069 PMC429360

[B12] Cha S. Min W. K. Seo H. S. (2024). Arabidopsis COP1 guides stomatal response in guard cells through pH regulation. Commun. Biol. 7, 150. doi: 10.1038/s42003-024-05847-w 38316905 PMC10844630

[B13] Chen K. Li G. J. Bressan R. A. Song C. P. Zhu J. K. Zhao Y. (2020). Abscisic acid dynamics, signaling, and functions in plants. J. Integr. Plant Biol. 62, 25–54. doi: 10.1111/jipb.12899 31850654

[B14] Cotelle V. Leonhardt N. (2019). ABA signaling in guard cells. Adv. Bot. Res. 92, 115–170. doi: 10.1016/bs.abr.2019.10.001

[B15] Demes E. Besse L. Cubero-Font P. Satiat-Jeunemaitre B. Thomine S. De Angeli A. (2020). Dynamic measurement of cytosolic pH and [NO_3_ ^-^] uncovers the role of the vacuolar transporter *AtCLCa* in cytosolic pH homeostasis. Proc. Nat. Acad. Sci. U.S.A. 117, 15343–15353. doi: 10.1073/pnas.2007580117 PMC7334523 32546525

[B16] Dreyer I. Li K. Riedelsberger J. Hedrich R. Konrad K. R. Michard E. (2022). Transporter networks can serve plant cells as nutrient sensors and mimic transceptor-like behavior. iScience 25, 104078. doi: 10.1016/j.isci.2022.104078 35378857 PMC8976136

[B17] Eisenach C. De Angeli A. (2017). Ion transport at the vacuole during stomatal movements. Plant Physiol. 174, 520–530. doi: 10.1104/pp.17.00130 28381500 PMC5462060

[B18] Felle H. H. (2001). pH: signal and messenger in plant cells. Plant Biol. 3, 577–591. doi: 10.1055/s-2001-19372

[B19] Felle H. H. Herrmann A. Hanstein S. Hückelhoven R. Kogel K. H. (2004). Apoplastic pH signaling in barley leaves attacked by the powdery mildew fungus *Blumeria graminis* f. sp. *hordei* . Mol. Plant Microbe Interact. 17, 118–123. doi: 10.1094/MPMI.2004.17.1.118 14714875

[B20] Gao D. Knight M. R. Trewavas A. J. Sattelmacher B. Plieth C. (2004). Self-reporting Arabidopsis expressing pH and Ca^2+^ indicators unveil ion dynamics in the cytoplasm and in the apoplast under abiotic stress. Plant Physiol. 134, 898–908. doi: 10.1104/pp.103.032508 15020753 PMC389913

[B21] Gehring C. A. Irving H. R. Parish R. W. (1990). Effects of auxin and abscisic acid on cytosolic calcium and pH in plant cells. Proc. Natl. Acad. Sci. U.S.A. 87, 9645–9649. doi: 10.1073/pnas.87.24.9645 11607135 PMC55229

[B22] Geilfus C. M. (2017). The pH of the apoplast: dynamic factor with functional impact under stress. Mol. Plant 10, 1371–1386. doi: 10.1016/j.molp.2017.09.018 28987886

[B23] Geilfus C. M. Mithöfer A. Ludwig-Müller J. Zörb C. Muehling K. H. (2015). Chloride-inducible transient apoplastic alkalinizations induce stomata closure by controlling abscisic acid distribution between leaf apoplast and guard cells in salt-stressed *Vicia faba* . New Phytol. 208, 803–816. doi: 10.1111/nph.13507 26096890

[B24] Gilroy S. Białasek M. Suzuki N. Górecka M. Devireddy A. R. Karpiński S.. (2016). ROS, calcium, and electric signals: Key mediators of rapid systemic signaling in plants. Plant Physiol. 171, 1606–1615. doi: 10.1104/pp.16.00434 27208294 PMC4936577

[B25] Gilroy S. Trewavas A. (1994). A decade of plant signals. BioEssays 16, 677–682. doi: 10.1002/bies.950160914

[B26] Gonugunta V. K. Srivastava N. Raghavendra A. S. (2009). Cytosolic alkalinization is a common and early messenger preceding the production of ROS and NO during stomatal closure by variable signals, including abscisic acid, methyl jasmonate and chitosan. Plant Signal. Behav. 4, 561–564. doi: 10.4161/psb.4.6.8847 19816133 PMC2688314

[B27] Grabov A. Blatt M. R. (1997). Parallel control of the inward-rectifier K^+^ channel by cytosolic free Ca^2+^ and pH in *Vicia* guard cells. Planta 201, 84–95. doi: 10.1007/BF01258684

[B28] Hager A. (2003). Role of the plasma membrane H^+^-ATPase in auxin-induced elongation growth: historical and new aspects. J. Plant Res. 116, 483–505. doi: 10.1007/s10265-003-0110-x 12937999

[B29] Han J. Burgess K. (2010). Fluorescent indicators for intracellular pH. Chem. Rev. 110, 2709–2728. doi: 10.1021/cr900249z 19831417

[B30] Han J. P. Köster P. Drerup M. M. Scholz M. Li S. Edel K. H.. (2019). Fine-tuning of RBOHF activity is achieved by differential phosphorylation and Ca^2+^ binding. New Phytol. 221, 1935–1949. doi: 10.1111/nph.15543 30320882

[B31] Hsu P. K. Dubeaux G. Takahashi Y. Schroeder J. I. (2021). Signaling mechanisms in abscisic acid-mediated stomatal closure. Plant J. 105, 307–321. doi: 10.1111/tpj.15067 33145840 PMC7902384

[B32] Hu J. An Y. Y. Cai C. Y. He S. S. Wang L. (2019). Cytoplasmic pH is involved in 5-aminolevulinic acid (ALA)-induced stomatal opening in apple leaves. Acta Hortic. Sinic. 46, 1869–1881. doi: 10.16420/j.issn.0513-353x.2018-0880

[B33] Huang A. X. She X. P. Zhao J. L. Zhang Y. Y. (2014). Inhibition of ABA-induced stomatal closure by fusicoccin is associated with cytosolic acidification-mediated hydrogen peroxide removal. Bot. Stud. 55, 33. doi: 10.1186/1999-3110-55-33 28510970 PMC5432956

[B34] Huang S. Shen L. Roelfsema M. R. G. Becker D. Hedrich R. (2023). Light-gated channelrhodopsin sparks proton-induced calcium release in guard cells. Science 382, 1314–1318. doi: 10.1126/science.adj9696 38096275

[B35] Inoue S. I. Kinoshita T. (2017). Blue light regulation of stomatal opening and the plasma membrane H^+^-ATPase. Plant Physiol. 174, 531–538. doi: 10.1104/pp.17.00166 28465463 PMC5462062

[B36] Irving H. R. Gehring C. A. Parish R. W. (1992). Changes in cytosolic pH and calcium of guard cells precede stomatal movements. Proc. Natl. Acad. Sci. U.S.A. 89, 1790–1794. doi: 10.1073/pnas.89.5.1790 11607281 PMC48538

[B37] Islam M. M. Hossain M. A. Jannat R. Munemasa S. Nakamura Y. Mori I. C.. (2010). Cytosolic alkalization and cytosolic calcium oscillation in Arabidopsis guard cells response to ABA and MeJA. Plant Cell Physiol. 51, 1721–1730. doi: 10.1093/pcp/pcq131 20739306

[B38] Kim T. H. Böhmer M. Hu H. Nishimura N. Schroeder J. I. (2010). Guard cell signal transduction network: advances in understanding abscisic acid, CO_2_, and Ca^2+^ signaling. Annu. Rev. Plant Biol. 61, 561–591. doi: 10.1146/annurev-arplant-042809-112226 20192751 PMC3056615

[B39] Kimura S. Kaya H. Hashimoto K. Wrzaczek M. Kuchitsu K. (2022). Quantitative analysis for ROS-producing activity and regulation of plant NADPH oxidases in HEK293T cells. Methods Mol. Biol. 2526, 107–122. doi: 10.1007/978-1-0716-2469-2_8 35657515

[B40] Kosegarten H. Grolig F. Wieneke J. Wilson G. Hoffmann B. (1997). Differential ammonia-elicited changes of cytosolic pH in root hair cells of rice and maize as monitored by 2’,7’-bis-(2-carboxyethyl)-5 (and -6)-carboxyfluorescein-fluorescence ratio. Plant Physiol. 113, 451–461. doi: 10.1104/pp.113.2.451 12223619 PMC158160

[B41] Li B. Chen Y. Tian S. (2022b). Function of pH-dependent transcription factor PacC in regulating development, pathogenicity, and mycotoxin biosynthesis of phytopathogenic fungi. FEBS J. 289, 1723–1730. doi: 10.1111/febs.15808 33751796

[B42] Li K. Grauschopf C. Hedrich R. Dreyer I. Konrad K. R. (2024). K^+^ and pH homeostasis in plant cells is controlled by a synchronized K^+^/H^+^ antiport at the plasma and vacuolar membrane. New Phytol. 241, 1525–1542. doi: 10.1111/nph.19436 38017688

[B43] Li K. Prada J. Damineli D. S. C. Liese A. Romeis T. Dandekar T.. (2021). An optimized genetically encoded dual reporter for simultaneous ratio imaging of Ca^2+^ and H^+^ reveals new insights into ion signaling in plants. New Phytol. 230, 2292–2310. doi: 10.1111/nph.17202 33455006 PMC8383442

[B44] Li W. Song T. Wallrad L. Kudla J. Wang X. Zhang W. (2019). Tissue-specific accumulation of pH-sensing phosphatidic acid determines plant stress tolerance. Nat. Plants. 5, 1012–1021. doi: 10.1038/s41477-019-0497-6 31451794

[B45] Li J. Yang Y. (2023). How do plants maintain pH and ion homeostasis under saline-alkali stress? Front. Plant Sci. 14. doi: 10.3389/fpls.2023.1217193 PMC10616311 37915515

[B46] Li Y. Zeng H. Xu F. Yan F. Xu W. (2022a). H^+^-ATPases in plant growth and stress responses. Ann. Rev. Plant Biol. 73, 495–521. doi: 10.1146/annurev-arplant-102820-114551 35231180

[B47] Liu J. Ji Y. Zhou J. Xing D. (2016). Phosphatidylinositol 3-kinase promotes activation and vacuolar acidification and delays methyl jasmonate-induced leaf senescence. Plant Physiol. 170, 1714–1731. doi: 10.1104/pp.15.00744 26739232 PMC4775102

[B48] Liu H. Song S. Zhang H. Li Y. Niu L. Zhang J.. (2022). Signaling transduction of ABA, ROS, and Ca^2+^ in plant stomatal closure in response to drought. Int. J. Mol. Sci. 23, 14824. doi: 10.3390/ijms232314824 36499153 PMC9736234

[B49] Lozano-Juste J. León J. (2010). Enhanced abscisic acid-mediated responses in *nia1nia2noa1-2* triple mutant impaired in NIA/NR- and AtNOA1-dependent nitric oxide biosynthesis in Arabidopsis. Plant Physiol. 152, 891–903. doi: 10.1104/pp.109.148023 20007448 PMC2815865

[B50] Ma Y. Niu J. (2017). The role of phytosphingosine-1-phosphate (Phyto-S1P) and its relationships with cytosolic pH and hydrogen peroxide (H_2_O_2_) during stomatal closure by darkness in broad bean. S. Afr. J. Bot. 108, 237–242. doi: 10.1016/j.sajb.2016.11.002

[B51] Ma Y. L. She X. P. Yang S. S. (2012). Sphingosine-1-phosphate (S1P) mediates darkness-induced stomatal closure through raising cytosol pH and hydrogen peroxide (H_2_O_2_) levels in guard cells in *Vicia faba* . Sci. China Life Sci. 55, 974–983. doi: 10.1007/s11427-012-4386-8 23090064

[B52] Ma Y. L. She X. P. Yang S. S. (2013). Cytosolic alkalization-mediated H_2_O_2_ and NO production are involved in darkness-induced stomatal closure in *Vicia faba* . Can. J. Plant Sci. 93, 119–130. doi: 10.4141/cjps2012-040

[B53] Martinière A. Gibrat R. Sentenac H. Dumont X. Gaillard I. Paris N. (2018). Uncovering pH at both sides of the root plasma membrane interface using noninvasive imaging. Proc. Nat. Acad. Sci. U.S.A. 115, 6488–6493. doi: 10.1073/pnas.1721769115 PMC6016826 29866831

[B54] Mathieu Y. Lapous D. Thomine S. Laurière C. Guern J. (1996). Cytoplasmic acidification as an early phosphorylation- dependent response of tobacco cells to elicitors. Planta 199, 416–424. doi: 10.1007/BF00195734

[B55] Meddya S. Meshram S. Sarkar D. R. S. Datta R. Singh S.. (2023). Plant Stomata: An unrealized possibility in plant defense against invading pathogens and stress tolerance. Plants (Basel Switzerland) 12, 3380. doi: 10.3390/plants12193380 37836120 PMC10574665

[B56] Meimoun P. Vidal G. Bohrer A. S. Lehner A. Tran D. Briand J.. (2009). Intracellular Ca^2+^ stores could participate to abscisic acid-induced depolarization and stomatal closure in *Arabidopsis thaliana* . Plant Signal. Behav. 4, 830–835. doi: 10.4161/psb.4.9.9396 19847112 PMC2802785

[B57] Merlot S. Leonhardt N. Fenzi F. Valon C. Costa M. Piette L.. (2007). Constitutive activation of a plasma membrane H^+^-ATPase prevents abscisic acid-mediated stomatal closure. EMBO J. 26, 3216–3226. doi: 10.1038/sj.emboj.7601750 17557075 PMC1914098

[B58] Miedema H. Assmann S. M. (1996). A membrane-delimited effect of internal pH on the K^+^ outward rectifier of *Vicia faba* guard cells. J. Memb. Biol. 154, 227–237. doi: 10.1007/s002329900147 8952952

[B59] Mirasole F. M. Nastasi S. P. Cubero-Font P. De Angeli A. (2023). Vacuolar control of stomatal opening revealed by 3D imaging of the guard cells. Sci. Rep. 13, 7647. doi: 10.1038/s41598-023-34273-x 37169939 PMC10175559

[B60] Monshausen G. B. Bibikova T. N. Messerli M. A. Shi C. Gilroy S. (2007). Oscillations in extracellular pH and reactive oxygen species modulate tip growth of *Arabidopsis* root hairs. Proc. Natl. Acad. Sci. U.S.A. 104, 20996–21001. doi: 10.1073/pnas.0708586104 18079291 PMC2409255

[B61] Murata Y. Mori I. C. Munemasa S. (2015). Diverse stomatal signaling and the signal integration mechanism. Ann. Rev. Plant Biol. 66, 369–392. doi: 10.1146/annurev-arplant-043014-114707 25665132

[B62] Oja V. Savchenko G. Jakob B. Heber U. (1999). pH and buffer capacities of apoplastic and cytoplasmic cell compartments in leaves. Planta 209, 239–249. doi: 10.1007/s004250050628 10436227

[B63] Pandey S. Zhang W. Assmann S. M. (2007). Roles of ion channels and transporters in guard cell signal transduction. FEBS Lett. 581, 2325–2336. doi: 10.1016/j.febslet.2007.04.008 17462636

[B64] Paradiso A. M. Tsien R. Y. Machen T. E. (1984). Na^+^-H^+^ exchange in gastric glands as measured with a cytoplasmic-trapped, fluorescent pH indicator. Proc. Natl. Acad. Sci. U.S.A. 81, 7436–7440. doi: 10.1073/pnas.81.23.7436 6095295 PMC392161

[B65] Pecherina A. Grinberg M. Ageyeva M. Zdobnova T. Ladeynova M. Yudintsev A.. (2021). Whole-plant measure of temperature-induced changes in the cytosolic pH of potato plants using genetically encoded fluorescent sensor Pt-GFP. Agriculture 11, 1131. doi: 10.3390/agriculture11111131

[B66] Pei D. Hua D. Deng J. Wang Z. Song C. Wang Y.. (2022). Phosphorylation of the plasma membrane H^+^-ATPase AHA2 by BAK1 is required for ABA-induced stomatal closure in Arabidopsis. Plant Cell 34, 2708–2729. doi: 10.1093/plcell/koac106 35404404 PMC9252505

[B67] Pittman J. K. Hirschi K. D. (2016). CAX-ing a wide net: Cation/H^+^ transporters in metal remediation and abiotic stress signalling. Plant Biol. 18, 741–749. doi: 10.1111/plb.12460 27061644 PMC4982074

[B68] Pottosin I. Velarde-Buendía A. M. Bose J. Fuglsang A. T. Shabala S. (2014). Polyamines cause plasma membrane depolarization, activate Ca^2+^-, and modulate H^+^-ATPase pump activity in pea roots. J. Exp. Bot. 65, 2463–2472. doi: 10.1093/jxb/eru133 24723394

[B69] Puli M. R. Raghavendra A. S. (2012). Pyrabactin, an ABA agonist, induced stomatal closure and changes in signalling components of guard cells in abaxial epidermis of *Pisum sativum* . J. Exp. Bot. 63, 1349–1356. doi: 10.1093/jxb/err364 22131162 PMC3276095

[B70] Raghavendra A. S. Ye W. Kinoshita T. (2023). pH as a signal and secondary messenger in plant cells. Front. Plant Sci. 14. doi: 10.3389/fpls.2023.1148689 PMC9928177 36798702

[B71] Rhaman M. S. Imran S. Rauf F. Khatun M. Baskin C. C. Murata Y.. (2020). Seed priming with phytohormones: an effective approach for the mitigation of abiotic stress. Plants (Basel) 10, 37. doi: 10.3390/plants10010037 33375667 PMC7824124

[B72] Ribeiro D. M. Desikan R. Bright J. Confraria A. Harrison J. Hancock J. T.. (2009). Differential requirement for NO during ABA-induced stomatal closure in turgid and wilted leaves. Plant Cell Environ. 32, 46–57. doi: 10.1111/j.1365-3040.2008.01906.x 19021879

[B73] Roelfsema M. R. G. Hedrich R. (2005). In the light of stomatal opening: new insights into 'the Watergate'. New Phytol. 167, 665–691. doi: 10.1111/j.1469-8137.2005.01460.x 16101906

[B74] Roelfsema M. R. G. Levchenko V. Hedrich R. (2004). ABA depolarizes guard cells in intact plants, through a transient activation of R- and S-type anion channels. Plant J. 37, 578–588. doi: 10.1111/j.1365-313x.2003.01985.x 14756768

[B75] Roos W. Viehweger K. Dordschbal B. Schumann B. Evers S. Steighardt J.. (2006). Intracellular pH signals in the induction of secondary pathways–the case of Eschscholzia californica. J. Plant Physiol. 163, 369–381. doi: 10.1016/j.jplph.2005.11.012 16413947

[B76] Salam M. A. Jammes F. Hossain M. A. Ye W. Nakamura Y. Mori I. C.. (2013). Two guard cell-preferential MAPKs, MPK9 and MPK12, regulate YEL signalling in *Arabidopsis* guard cells. Plant Biol. 15, 436–442. doi: 10.1111/j.1438-8677.2012.00671.x 23043299

[B77] Schulte A. Lorenzen I. Böttcher M. Plieth C. (2006). A novel fluorescent pH probe for expression in plants. Plant Methods 2, 7. doi: 10.1186/1746-4811-2-7 16600023 PMC1475855

[B78] Seidel T. (2022). The plant V-ATPase. Front. Plant Sci. 13. doi: 10.3389/fpls.2022.931777 PMC9280200 35845650

[B79] Sobahan M. A. Akter N. Okuma E. Uraji M. Ye W. Mori I. C.. (2015). Allyl isothiocyanate induces stomatal closure in *Vicia faba* . Biosci. Biotechnol. Biochem. 79, 1737–1742. doi: 10.1080/09168451.2015.1045827 26027691

[B80] Suhita D. Raghavendra A. S. Kwak J. M. Vavasseur A. (2004). Cytoplasmic alkalization precedes reactive oxygen species production during methyl jasmonate- and abscisic acid-induced stomatal closure. Plant Physiol. 134, 1536–1545. doi: 10.1104/pp.103.032250 15064385 PMC419829

[B81] Takemiya A. Shimazaki K. (2010). Phosphatidic acid inhibits blue light-induced stomatal opening via inhibition of protein phosphatase 1. Plant Physiol. 153, 1555–1562. doi: 10.1104/pp.110.155689 20498335 PMC2923901

[B82] Trinh M. D. L. Masuda S. (2022). Chloroplast pH homeostasis for the regulation of photosynthesis. Front. Plant Sci. 13. doi: 10.3389/fpls.2022.919896 PMC9174948 35693183

[B83] van Meeteren U. Kaiser E. Malcolm Matamoros P. Verdonk J. C. Aliniaeifard S. (2020). Is nitric oxide a critical key factor in ABA-induced stomatal closure? J. Exp. Bot. 71, 399–410. doi: 10.1093/jxb/erz437 31565739 PMC6913703

[B84] Wei A. Fu B. Wang Y. Zhai X. Xin X. Zhang C.. (2015). Involvement of NO and ROS in sulfur dioxide induced guard cells apoptosis in *Tagetes erecta* . Ecotoxicol. Environ. Saf. 114, 198–203. doi: 10.1016/j.ecoenv.2015.01.024 25645141

[B85] Wilkins K. A. Bosch M. Haque T. Teng N. Poulter N. S. Franklin-Tong V. E. (2015). Self-incompatibility-induced programmed cell death in field poppy pollen involves dramatic acidification of the incompatible pollen tube cytosol. Plant Physiol. 167, 766–779. doi: 10.1104/pp.114.252742 25630437 PMC4347735

[B86] Wong J. H. Spartz A. K. Park M. Y. Du M. Gray W. M. (2019). Mutation of a conserved motif of PP2C.D phosphatases confers SAUR immunity and constitutive activity. Plant Physiol. 181, 353–366. doi: 10.1104/pp.19.00496 31311832 PMC6716246

[B87] Yang J. Li C. Kong D. Guo F. Wei H. (2020). Light-mediated signaling and metabolic changes coordinate stomatal opening and closure. Front. Plant Sci. 11. doi: 10.3389/fpls.2020.601478 PMC7746640 33343603

[B88] Ye W. Koya S. Hayashi Y. Jiang H. Oishi T. Kato K.. (2021). Identification of genes preferentially expressed in stomatal guard cells of *Arabidopsis thaliana* and involvement of the aluminum-activated malate transporter 6 vacuolar malate channel in stomatal opening. Front. Plant Sci. 12. doi: 10.3389/fpls.2021.744991 PMC8531587 34691123

[B89] Yemelyanov V. V. Chirkova T. V. Shishova M. F. Lindberg S. M. (2020). Potassium efflux and cytosol acidification as primary anoxia-induced events in Wheat and Rice seedlings. Plants (Basel). 9, 1216. doi: 10.3390/plants9091216 32948036 PMC7570052

[B90] Zhang J. Chen X. Song Y. Gong Z. (2024). Integrative regulatory mechanisms of stomatal movements under changing climate. J. Integr. Plant Biol. 66, 368–393. doi: 10.1111/jipb.13611 38319001

[B91] Zhang X. Dong F. C. Gao J. F. Song C. P. (2001). Hydrogen peroxide-induced changes in intracellular pH of guard cells precede stomatal closure. Cell Res. 11, 37–43. doi: 10.1038/sj.cr.7290064 11305323

[B92] Zhang H. Niu X. Liu J. Xiao F. Cao S. Liu Y. (2013). RNAi-directed downregulation of vacuolar H^+^-ATPase subunit a results in enhanced stomatal aperture and density in rice. PLoS One 8, e69046. doi: 10.1371/journal.pone.0069046 23894405 PMC3718813

[B93] Zhou J. Y. Hao D. L. Yang G. Z. (2021). Regulation of cytosolic pH: The contributions of plant plasma membrane H^+^-ATPases and multiple transporters. Int. J. Mol. Sci. 22, 12998. doi: 10.3390/ijms222312998 34884802 PMC8657649

[B94] Zhu Y. Ge X. M. Wu M. M. Li X. He J. M. (2014). The role and interactions of cytosolic alkalization and hydrogen peroxide in ultraviolet B-induced stomatal closure in Arabidopsis. Plant Sci. 215-216, 84–90. doi: 10.1016/j.plantsci.2013.11.010 24388518

